# *In vitro* inhibition of *Mycobacterium tuberculosis β*-carbonic anhydrase 3 with Mono- and dithiocarbamates and evaluation of their toxicity using zebrafish developing embryos

**DOI:** 10.1080/14756366.2019.1683007

**Published:** 2019-10-30

**Authors:** Ashok Aspatwar, Milka Hammaren, Mataleena Parikka, Seppo Parkkila, Fabrizio Carta, Murat Bozdag, Daniela Vullo, Claudiu T. Supuran

**Affiliations:** a Faculty of Medicine and Health Technology, Tampere University, Tampere, Finland; b Oral and Maxillofacial Unit, Tampere University Hospital, Tampere, Finland; c Fimlab Ltd, Tampere University Hospital, Tampere, Finland; d NEUROFARBA Department, Sezione di Scienze Farmaceutiche e Nutraceutiche, University of Florence, Sesto Fiorentino (Florence), Italy

**Keywords:** *β*-carbonic anhydrase, monothiocarbamates (MTCs), dithiocarbamate (DTCs), in vitro inhibition, zebrafish larvae

## Abstract

We investigated a panel of 14 compounds belonging to the monothiocarbamate (**MTC**) and dithiocarbamate (**DTC**) series against the β-carbonic anhydrase 3 (*β*-CA3) of *Mycobacterium tuberculosis* (Mtb). We also evaluated all compounds for toxicity using 1–5-day post fertilisation zebrafish embryos. 11 out of the 14 investigated derivatives showed effective nanomolar or submicromolar *in vitro* inhibition against the *β*-CA3 (K_I_s 2.4–812.0 nM), and among them four **DTCs** of the series (**8–10** and **12**) showed very significant inhibition potencies with K_I_s between 2.4 and 43 nM. Out of 14 compounds screened for toxicity and safety 9 compounds showed no adverse phenotypic effects on the developing zebrafish larvae at five days of exposure. The results of *in vitro* inhibition and the toxicological evaluation of our study suggest that 5 compounds are suitable for further *in vivo* preclinical characterisation in zebrafish model.

## Introduction

Mtb is a highly infectious microbial species that causes tuberculosis (TB) in humans. The latest World Health Organisation report on infectious diseases estimated that annually, 10 million people develop TB and 1.6 million die from the disease^1^. Quite worryingly, the same study reported >550 000 infections to be caused by rifampin-resistant strains that are unresponsive to this first-line drug against Mtb. Among these strains, 80% were multidrug-resistant (MDR-TB)^1^. Therefore, there is an urgent need for antibiotics targeting novel physiological pathways of Mtb^2–4^.

**Figure 1.**
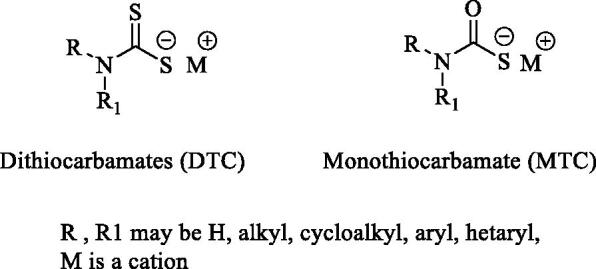
General structures of dithiocarbamates (**DTC**) and monothiocarbamates (**MTC**).

Mycobacteria encode for at least three zinc-containing metalloenzyme carbonic anhydrases (CAs, EC 4.2.1.1) that belong to the β-CA gene family^1^^,^^2^. Since the primary function of *β*-CAs in mycobacteria is to reversibly catalyse the hydration of CO_2_ to generate HCO_3_^−^ and H^+^ ions^5^^,^^6^, such enzymes are involved in a multitude of physiological processes closely related to pH homeostasis, biosynthetic processes as well as adaption to the environments^3^^,^^7–10^. In addition, mycobacterial β-CAs have been shown to be essential for the transport of extracellular DNA (eDNA)^11^. It was also reported that the administration of the non-selective CA Inhibitor (CAI) ethoxzolamide (**EZA**) reduced transport of eDNA and affected the formation of biofilms in non-tuberculous mycobacteria (NTM)^11^. Another study conducted on *Mycobacterium tuberculosis* (Mtb) strains, demonstrated that **EZA** inhibited the two-component PhoPR regulon as well as the Esx-1 protein secretion system, which are fundamental for the virulence of the bacterium^12^. **EZA** also showed efficacy in infected macrophages and mice suggesting that *β*-CAs perform very important roles in mycobacterial infections^12^, and hence present themselves as potential drug targets. The three Mtb β-CAs (i.e. β-CA1, *β*-CA2 and *β*-CA3) are encoded by Rv1284, Rv3588c and Rv3273 genes, respectively^8^^,^^13–16^. Among the compounds tested *in vitro* for their inhibition properties against such enzymes are the classical CAIs of the sulphonamide type^17^^,^^18^^,^ and the recently reported dithio- and monothiocarbamates^19^^,^^20^ (**DTCs** and **MTCs**) whose general structures are shown in Figure 1.

Both **DTCs** and **MTCs** have been explored, with good outcomes, against several CAs from pathogens expressing *α*-, *β*-, and *δ*-CA enzymes^19–23^. A study conducted in our laboratory showed that a **DTC**-based compound (i.e. derivative **12** in this study) not only effectively inhibited the β-CAs of Mtb but also impaired the growth of *Mycobacterium marinum,* a close relative of Mtb, in zebrafish larvae^2^. The data obtained by means of *in vitro* and *in vivo* experiments involving **DTCs** and **MTCs** encouraged us to further investigate similar compounds against the Mtb *β*-CA3 with the aim to find new potential inhibitors targeting mycobacterial *β*-CAs^2^^,^^21^.

## Materials and methods

### Chemistry

Compounds **1-14** considered in this manuscript were synthesised according to the procedures previously reported by some of us, and they were properly characterised by means of 1H-NMR, 13 C-NMR, and mass spectra analysis^24–27^.

### In vitro carbonic anhydrase inhibition assay

The CA-catalyzed CO_2_ hydration activity was assayed on an Applied Photophysics stopped-flow instrument using phenol red (at a concentration of 0.2 mM) as a pH indicator with 20 mM Hepes (pH 7.5) as the buffer, 20 mM Na_2_SO_4_, and following the initial rates of the CA-catalyzed CO_2_ hydration reaction for a period of 10 − 100 s and working at the maximum absorbance of 557 nm^28–30^. The CO_2_ concentrations ranged from 1.7 to 17 mM. For each inhibitor six traces of the initial 5 − 10% of the reaction have been used in order to determine the initial velocity. The uncatalyzed reaction rates were determined in the same manner and subtracted from the total observed rates. Stock solutions of inhibitor (0.1 mM) were prepared in distilled water, and dilutions up to 0.01 nM were prepared. Solutions containing the inhibitor and enzyme were preincubated for 15 min at room temperature prior to assay in order to allow the formation of the E − I complex. The inhibition constants were obtained as nonlinear least-squares protocols using PRISM 3^28–30^ and are the mean from at least three different measurements. All hCAs were recombinant ones and were obtained in house^28–30^.

## Toxicity evaluation

### Inhibitors

Compounds **1–14** were either dissolved in Embryonic medium [5.0 mM NaCl, 0.17 mM KCl, 0.33 mM CaCl_2_, 0.33 mM MgSO_4_, and 0.1% w/v Methylene Blue (Sigma-Aldrich, Germany)] or in dimethyl sulfoxide (DMSO) (Sigma-Aldrich, St. Louis, MO) to prepare 100 mM stock solutions. Before the start of each experiment, the series of dilutions were made from the above stock in the embryonic medium.

### Maintenance of zebrafish

The wild type adult zebrafish (AB strains) were maintained at 28.5 °C. 3–5 pairs of male and female fish were moved to breeding tanks overnight^31^. Next morning, 1–2-h post fertilisation (hpf), embryos were collected in a sieve and rinsed with embryonic medium, and the collected embryos were maintained in an incubator at 28.5 °C overnight^31^. The toxicity evaluation studies of the inhibitors were initiated with fish embryos 24-hpf. All the fish experiments were performed at the zebrafish core facility of Tampere University according to the protocol used in our laboratory^32^.

### Ethical statement

The research unit at Tampere University has an established zebrafish core facility authorised by the National Animal Experiment Board (ESAVI/7975/04.10.05/2016). The experiments using developing zebrafish embryos were performed according to the Provincial Government of Eastern Finland Province Social and Health Department Tampere Regional Service Unit protocol # LSLH-2007–7254/Ym-23. Care was taken to ameliorate suffering by euthanizing the 5 dpf larvae by prolonged immersion in a petri dish containing an overdose of Tricaine (Sigma-Aldrich, St. Louis, MO) before fixing in buffered formaldehyde for histochemical analysis.

### Determination of median lethal concentration 50 (LC_50_)

The LC_50_ values for all the MTC and DTC compounds were determined using 24-hpf embryos with 10–12 different concentrations for each compound. For every concentration of the inhibitor, we used 30 24-hpf embryos^31^^,^^32^. For each compound, the fish were exposed to different concentrations of the inhibitors rangeing from 5 μM to 2.5 mM. Dose response curve (DRC) was calculated using DRM of the DRC R package^33^. The control group larvae constituted an equal number of larvae not treated with any inhibitor compound and the larvae that were treated with 1% of DMSO. Toxicological evaluation studies were performed in 24-well plates (Corning V R Co-star V R cell culture plates). One or two 24-hpf embryos in were placed per well in 1 mL of embryonic medium containing a diluted inhibitor. A minimum of three sets of experiments were carried out for each inhibitor. Mortality of the larvae was checked every 24 h until 5 days after exposure to the inhibitors.

### Phenotypic analysis of control and inhibitor treated larvae

After exposure to the inhibitors, we evaluated the effects of these inhibitors on the zebrafish larvae and analysed eight phenotypic parameters: (1) mortality, (2) hatching, (3) oedema, (4) swimming pattern, (5) yolk sack utilisation, (6) heartbeat, (7) body shape, and (8) swim bladder development. The images of the developing larvae were taken using a Lumar V1.12 microscope attached to a camera (Carl Zeiss MicroImaging GmbH, Göttingen, Germany). The images were analysed with AxioVision software versions 4.7 and 4.8 as described in our standard protocol for assessment of toxicity and safety of the chemical compounds^32^.

### Swim pattern analysis

The swim pattern of the zebrafish larvae was studied after 5 days of exposure to these inhibitors. For the analyses of swim pattern, about 10–15 zebrafish larvae were placed in a 35 mm X 15 mm petri dish containing embryonic medium and the larvae were allowed to settle in the petri dish for 1 min. The movement of the zebrafish larvae was observed under the microscope for 1 min. The swim patterns were compared with the control group zebrafish larvae that were not treated with any inhibitor.

### Histological studies

The histochemical analyses were done to assess the effect of inhibitors on the morphology of tissues of the larvae that were treated with different concentrations of inhibitors. The control group larvae were treated with embryonic medium alone or with 1% DMSO in embryonic medium. After 5 days of treatment the larvae were washed with phosphate buffered saline (PBS) and immersed in excess amounts of Tricaine to anaesthetize them. The larvae were transferred to a 1.5 mL microcentrifuge tube and fixed in buffered formaldehyde (4% formaldehyde solution, pH 6.9) in PBS for 3 h at room temperature or overnight at 4 °C. After the fixation, the larvae were transferred to 70% ethanol and stored at 4 °C before embedding in paraffin. The samples were then sectioned into 5 μm thin slices for the histochemical staining. The sections were deparaffinized in xylene, rehydrated in an alcohol series, and stained with Mayer's Haematoxylin and Eosin Y (both from Sigma-Aldrich). After dehydration, the slides were mounted with Entellan^®^ Neu (Merck; Darmstadt, Germany). The slides containing the tissues were examined for morphological changes and photographed using a Nikon Microphot microscope (Nikon Microphot- FXA, Japan). All the procedures were carried out at room temperature unless stated otherwise.

## Results and discussion

### Chemistry

**Figure 2.**
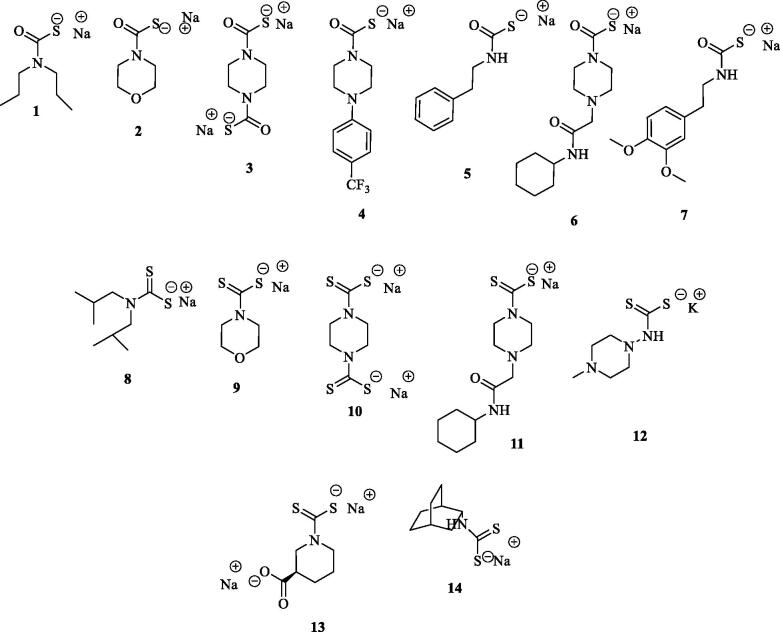
Chemical structures of **MTCs 1-7** and **DTCs 8-14**.

The main structural differences among the compounds tested in this study are related to the metal binding moieties of CAs. The compounds included both monothiocarbamates (**MTCs**) **1–7** and dithiocarbamates (**DTCs**) **8–14** (Figure 2).

In order to efficiently explore the inhibition potencies of the **MTC** and **DTC** binding moieties on Mtb *β*-CA3, we decided to consider small size and lined-shaped scaffolds lacking any bulky substitution. Such an approach was also justified by the narrower size of the β CA’s cavities when compared to the *α* ones^34^. As reported in Figure 2 almost all compounds responded to such a rule and the only exceptions were the 3-carboxy substituted piperazine **13** and the bicyclo[2.2.2]octan-2-amine **14**.

### CA inhibition

Compounds **1–14** were tested *in vitro* for their inhibitory properties against the Mtb *β*-CA3 and the data are reported in Table 1.

**Table 1.** Inhibition data of Mtb *β*-CA3 and human CA isoforms hCA I and II for compounds **1–14**, determined by the stopped-flow CO_2_ hydrase assay^35^, using acetazolamide (**AAZ**) as a standard drug.

	*K_I_* (nM)^a^		
Compounds	Mtb *β*-CA3	hCA I	hCA II
**1**	558.6	>2000^26^	46.7^26^
**2**	747.6	569^26^	>2000^26^
**3**	812.0	876^26^	22.4^26^
**4**	83.3	>2000^26^	43.6^26^
**5**	93.0	>2000^26^	43.7^26^
**6**	780.7	949^26^	45.9^26^
**7**	97.9	891^26^	26.7^26^
**8**	43.0	0.97^25^	0.95^25^
**9**	2.4	0.88^25^	0.95^25^
**10**	8.0	12.6^25^	0.92^25^
**11**	>2000	415^27^	67.2^27^
**12**	2.6	33.5^25^	33.0^25^
**13**	>2000	496^27^	80.5^27^
**14**	>2000	494^27^	48.7^27^
**AAZ**	104	250.0	12.0

^a^ Mean from 3 different assays, by a stopped flow technique (errors were in the range of ± 5–10% of the reported values).

The kinetic data reported in Table 1 clearly showed that among the **MTCs** tested the derivative **4** was the most potent compound in inhibiting the Mtb *β*-CA3 with a K_I_ value of 83.3 nM, followed by the phenylethyl **5** (K_I_ 93.0 nM) and its 3,4-dimethoxyphenylethyl derivative **7** (K_I_ 97.9 nM), and thus slightly more potent than the reference CAI **AAZ** (K_I_ 104 nM). All the other remaining **MTCs** (i.e. **1–3** and **6**) were high nanomolar range inhibitors of the *β*-CA3 with K_I_ values spanning between 558.6 and 812.0 nM. Interestingly **MTCs 4, 5** and **7** were ineffective inhibitors of the hCA I (K_I_s >2000 for compounds **4** and **5**, 891 nM for **7** respectively). Conversely, compounds **4**, **5** and **7** showed good inhibition data on hCA II being up to 3.7 fold more potent (K_I_s of 43.6, 43.7 and 26.7 nM, respectively). An analogous kinetic trend was observed for the remaining **MTCs 1**, **3** and **6** which were quite effective inhibitors of the hCA II over the hCA I isoform. The only exception is represented by the **MTC** based morpholine **2** which showed high nanomolar K_I_ value on the hCA I (K_I_ 569 nM) and no effects on the hCA II isoform (K_I_ >2000).

As for the isosteric **DTC** derivatives **8–14** a rather different kinetic profile was observed. For instance, the morpholine **DTC 9** was the most potent inhibitor within the series against Mtb *β*-CA3 (K_I_ of 2.4 nM) and quite interestingly it was 311.5 fold more potent when compared to its MTC analogous compound **2** (see Table 1). A similar kinetic trend was reported also for the bis-piperazine derivative **DTC 10** in comparison to its **MTC** counterpart **3** with the former being 101.5 fold more potent on the *β*-CA3 (K_I_s of 8.0 and 812.0 nM, respectively). An opposite kinetic trend was reported for the **DTC 11** which was ineffective on the *β*-CA3, whereas its **MTC** derivative **6** was a high nanomolar inhibitor (K_I_s of >2000 and 780.7 nM, respectively). Among the **DTC**s reported in this study, the *N*-methylpiperazine **12** was the second most potent inhibitor against the *β*-CA3 just after the morpholine derivative **9** (K_I_ of 2.6 and 2.4 nM, respectively). The structural differences between **9** and **12** (i.e. an oxygen and an *N*-methyl moiety at 4-position of the scaffold, respectively) did not affect the inhibition potencies against the *β*-CA3, whereas they proved crucial for the inhibition of the hCAs I and II. As reported in Table 1 the morpholine derivative **9** was a sub-nanomolar inhibitor of the hCAs reported (K_I_s of 0.88 and 0.95 nM for the hCA I and II, respectively), whereas the *N*-methyl piperazine **12** was a medium potency inhibitor (K_I_s of 33.5 and 33.5 nM, respectively), that makes the latter more selective against the *β*-CA3 isoform. Interestingly, the substitution of the alkyl ring in **9** or **12** with a bis-alkyl chain, as in compound **8**, or its modification to produce **13** and **14**, resulted in detrimental reductions for the inhibition potency against the *β*-CA3 (see Table 1).

### Evaluation of safety and toxicity

#### Determination of inhibitor LC_50_ concentrations

The lethal concentrations of the **MTC** and **DTC 1–14** inhibitors were tested on developing zebrafish embryos. The lowest concentration that caused death of half of the embryos was reported for the **DTC 8** (i.e. LD_50_ 2** **μM), which was therefore considered highly toxic and not suitable for further testing in our studies. The LC_50_ values of all the compounds are shown in Table 2.

**Table 2.** LC_50_ of the CAIs **1–14**.

Compounds	LC_50_ dose	*In vivo* studies (μM)^a^
**1**	1 mM	400
**2**	1 mM	125
**3**	2 mM	500
**4**	125 μM	35
**5**	1 mM	400
**6**	2 mM	500
**7**	2 mM	500
**8**	2 μM	1
**9**	125 μM	18
**10**	2 mM	500
**11**	1 mM	250
**12**	600 μM	300^b^
**13**	1 mM	500
**14**	25 μM	5

^a^ The concentrations do not induce any phenotypic defects in zebrafish larvae at 5** **days of exposure and safe for inhibition studies of *M. marinum* in zebrafish^2^.

^b^ The compound **12** (Fc14-584b) is screened for toxicity and for *in vivo* inhibition studies as reported earlier^2^.

#### Phenotypic analyses of zebrafish larvae treated with inhibitor compounds

**Figure 3.**
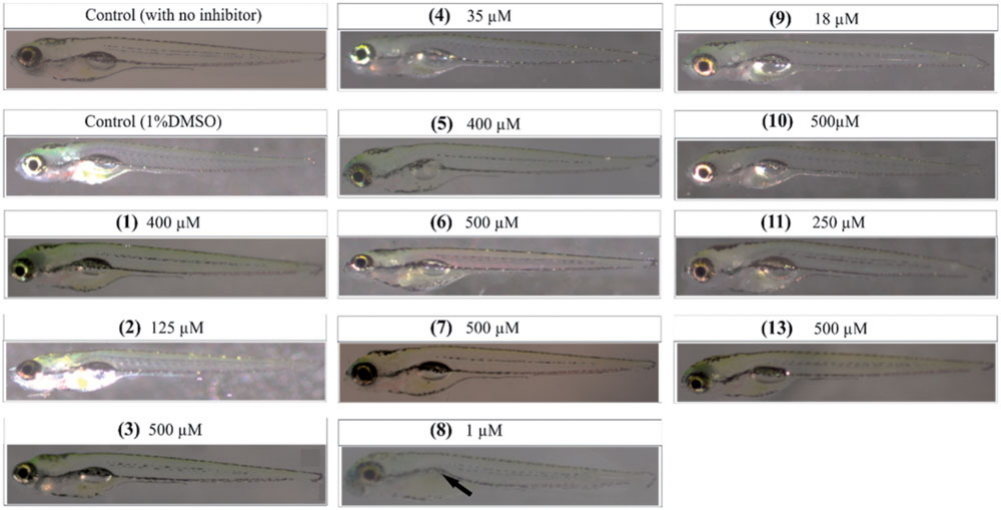
Images of zebrafish larvae treated with different inhibitors. The identification number of each compound is in parenthesis. The images of the zebrafish larvae after 5** **days of exposure to inhibitors that are considered safe for *in vivo* use to inhibit *M. marinum* growth in larvae^2^. The concentrations of the **MTCs** and **DTCs** shown here are the highest ones generally not inducing phenotypic defects at the end of 5** **days of exposure. For compound 8, even the lowest concentration used caused the absence of swim bladder (arrow), suggesting that this compound is not suitable for further characterization^2^.

To assess the toxic effects of the inhibitors on 5-day-old zebrafish during development after 4-day exposure to the compounds, we analysed seven observable phenotypic parameters (Table 3) using a stereo microscope and recorded the observations for each group. The zebrafish treated with CAIs were compared with the control groups not treated with any compound or with 1% DMSO. The maximum concentration of each compound that does not induce any phenotypic changes in the larvae at 5** **days of exposure was considered as safe (Table 2) and can be used for in vivo inhibition of M. marinum in zebrafish larvae^2^. The images in Figure 3 show representative larvae exposed to **MTCs** and **DTCs** in which no apparent phenotypic defects were observed except the compound **8** that showed defects in the development of swim bladder (Figure 3, arrow). **DTC 12** that inhibited Mtb β-CAs *in vitro* efficiently in our earlier studies^36^, showed similar results and was used for *in vivo* inhibition of *M. marinum* in zebrafish studies^1^.

**Table 3.** Effect of inhibitors on phenotypic parameters of the larvae 5** **days of after exposure^a^^,^^b^.

Compounds	Hatching	Oedema	Heartbeat	Yolk sac	Body shape	Swim bladder
**1**	100	0	0	0	6	0
**2**	100	5	4	5	5	0
**3**	100	0	0	0	2	0
**4**	100	0	3	0	0	0
**5**	100	2	0	3	1	0
**6**	100	0.7	0	0	0.7	0.7
**7**	100	0	0	1	1	0
**8**	80	10	100	0	8	22
**9**	0	NA	NA	NA	NA	NA
**10**	100	0	0	0	0	0
**11**	100	0	0	0	0	0
**12**^c^	NA	NA	NA	NA	NA	NA^2^
**13**	100	2	0	2	4	0
**14**	100	12	7	8	10	14

NA: not applicable.

^a^ The parameters were assessed at LC50 at 5** **day of exposure to the compounds.

^b^ The values shown are in percent of larvae.

^c^ The compound was screened for toxicity in our previous studies.

Further analyses of the embryos to assess the toxicity of compounds at the concentrations that are considered as safe (Table 2) on the observable phenotypic parameters showed that some of the compounds had no significant effects on any of the parameters assessed (Table 3). The compounds that were considered toxic, showed different level of toxicity by inducing high mortality, problem with hatching, oedema, heartbeat, yolk sac utilisation, body shape, and swim bladder development (Figure 3) as shown in Table 3.

#### Histochemical analysis

We analysed the sections of the zebrafish that were exposed to the inhibitors which did not have any significant effect on the phenotypic parameters (Table 2) of the larvae at the end of 5** **days of exposure to the inhibitors. The histologically stained sections of the inhibitor-treated larvae were compared with the stained sections of the control group larvae. None of the inhibitors showed any morphological changes of tissues of the zebrafish larvae exposed to the **MTCs** or **DTCs** (data not shown). The results of the histological examination suggest that these inhibitors cause no damage to the internal tissues at the concentrations tested and can be used for further characterisation.

#### Swim pattern analysis of larvae exposed to the inhibitors

Zebrafish embryos are easily affected by chemical compounds compared to adult zebrafish or other animal models and are hence suitable model organisms for assessing the subtle toxic effects of chemicals^32^^,^^37^. In this study, we further assessed the subtle toxic effects of the inhibitors by analysing the swim patterns of the larvae during exposure to the inhibitors at concentration that are considered as safe shown in Table 2. The swim pattern analysis showed that 11 out of 14 compounds showed no abnormal or ataxic movement of the larvae that were exposed to the inhibitors (data now shown). Therefore, most of the compounds that efficiently inhibit Mtb *β*-CA3 show no or minimal toxicity at relevant concentrations and are thus safe for further characterisation *in vivo*^2^^,^^32^ (Table 4).

**Table 4.** The compounds that inhibit Mtb *β*-CA3 efficiently and show minimal toxicity.

Compounds	*K_I_* (nM)^a^	Safe concentration (μM)^b^
**1**	558.6	400
**3**	812.0	500
**5**	93.0	400
**7**	97.9	500
**10**	8.0	500

^a^ Inhibition of β-CA3 *in vitro*.

^b^ The concentration that can be used for *in vivo* studies.

## Conclusions

In this study, we investigated a series of 14 CAIs belonging to the MTC and DTC zinc binding moieties possessing a variety of scaffolds and intended as inhibitors of the *β*-CA3 from *M. tuberculosis.* All compounds were investigated *in vitro* for their inhibition potencies against Mtb *β*-CA3 and compared to the human hCA I and II. The Mtb *β*-CA3 was efficiently inhibited by 11 of these derivatives with K_I_s in the range of 2.4–812** **nM. We evaluated these compounds for their toxic effects on 1–5 dpf zebrafish larvae. The toxicological studies showed that the compounds **1, 3, 5, 7,** and **10** exhibited minimal toxicity and were considered as safe for further characterisation *in vivo*. The results of the *in vitro* inhibition and toxicological evaluation studies showed that 5 compounds can be used for *in vivo* inhibition of the *M. marinum* growth in zebrafish larvae for further preclinical characterisation.
